# Effects of Heavy-Metal-Sludge Sintered Aggregates on the Mechanical Properties of Ultra-High-Strength Concrete

**DOI:** 10.3390/ma18143422

**Published:** 2025-07-21

**Authors:** Weijun Zhong, Sheng Wang, Yue Chen, Nan Ye, Kai Shu, Rongnan Dai, Mingfang Ba

**Affiliations:** 1 Ningbo Power Design Institute Co., Ltd., Ningbo 315020, China; 17280852161@163.com (W.Z.); nanye2332025@163.com (N.Y.); kaishu2025@163.com (K.S.); dairongnan@163.com (R.D.); 2 School of Civil & Environmental Engineering and Geography Science, Ningbo University, Ningbo 315211, China; 2211110042@nbu.edu.cn (S.W.); 2311110042@nbu.edu.cn (Y.C.)

**Keywords:** ultra-high-strength concrete, heavy-metal sludge, sintered slag aggregate, workability, mechanical properties, microstructural characteristics

## Abstract

To investigate the effects of heavy-metal-sludge sintered aggregates on the workability, mechanical properties, and fracture toughness of ultra-high-strength concrete (UHSC), this study systematically evaluated the influence of different aggregate replacement ratios and particle gradations on the fluidity, flexural strength, compressive strength, and fracture energy of UHSC. Microstructural characterization techniques including SEM, XRD, TG, and FTIR were employed to analyze the hydration mechanism and interfacial transition zone evolution. The results demonstrated the following: Fluidity continuously improved with the increase in the sintered aggregate replacement ratio, with coarse aggregates exhibiting the most significant enhancement due to the “ball-bearing effect” and paste enrichment. The mechanical properties followed a trend of an initial increase followed by a decrease, peaking at 15–20% replacement ratio, at which flexural strength, compressive strength, and fracture energy were optimally enhanced; excessive replacement led to strength reduction owing to skeletal structure weakening, with coarse aggregates providing superior improvement. Microstructural analysis revealed that the sintered aggregates accelerated hydration reactions, promoting the formation of C-S-H gel and Ca(OH)_2_, thereby densifying the ITZ. This study identified 15–20% of coarse sintered aggregates as the optimal replacement ratio, which synergistically improved the workability, mechanical properties, and fracture toughness of UHSC.

## 1. Introduction

Ultra-high-strength concrete (UHSC) has broad application prospects due to its advantages such as light weight, high strength, and excellent durability, which align with modern construction trends towards taller buildings, larger spans, and lighter structures [1,2,3,4]. However, the high consumption of Portland cement and natural aggregates in traditional UHSC formulations leads to increased costs and environmental burdens, especially concerning carbon emissions and the sustainability of resources. This issue urgently requires a solution [1]. In recent years, some scholars have investigated the use of industrial solid waste to produce aggregates as a replacement for natural aggregates in the preparation of green, low-carbon UHSC [5]. Such alternatives offer benefits like low cost, low shrinkage, ultra-high strength, and high durability [6], which makes them promising for future applications. For instance, Yang et al. [7] developed environmentally friendly UHSC using lithium slag, and their results indicated that lithium slag contributed to internal curing effects and volcanic ash reactions that enhanced the strength development of UHSC. Zhu et al. [8] used chromium iron slag as an aggregate to replace river sand in UHSC production and found that specific sizes of chromium iron slag contributed to better interface bonding, while reducing the maximum particle size led to poorer processing performance of UHSC. Zhao et al. [9] prepared UHSC using calcined coal gangue aggregates, demonstrating that active calcined coal gangue aggregates could compact the interfacial transition zone and reduce the porosity of UHSC, allowing for compressive strength of up to 143.1 MPa. Khan et al. [10] prepared UHSC by incorporating recycled glass powder and electric arc furnace slag, and their optimized mixture achieved a compressive strength threshold of 150 MPa as per ACI-239 standards [11].

It is noteworthy that the main chemical components of heavy-metal sludge (such as SiO_2_, Al_2_O_3_, etc.) are similar to those found in clay or shale [12], indicating potential pozzolanic activity. Additionally, heavy-metal sludge typically has a small particle size and a large specific surface area, which makes it an ideal raw material for producing slag after sintering treatment [13], and it shows potential as an ideal aggregate. Jankovský et al. [14] found that plasma gasification slag contaminated by heavy metals can serve as aggregate in magnesium oxychloride cement (MOC). Its incorporation enhances water resistance and mitigates the primary inherent limitation of MOC-based materials. Ma et al. [15] incorporated sintered heavy-metal sludge into cement-based materials, demonstrating that this sintered sludge improved pore structure and strengthened the interfacial transition zone, resulting in a 56% increase in the strength of the cement-based materials. Shen et al. [16] added sintered heavy-metal sludge to modified magnesium sulfate (MMOS) cement, and their findings indicated that this sludge promoted the early strength development of MMOS, with compressive strength and flexural strength surpassing the minimum requirements of GB/T 1596-2017 [17] by 28 days. However, to date, there has been no research investigating the use of sintered heavy-metal-sludge slag in the preparation of UHSC, nor has there been a comprehensive feasibility assessment of its potential as a replacement aggregate.

In summary, this paper aims to investigate the effects of different replacement ratios of heavy-metal-sludge sintered slag as aggregate and particle gradation on the workability, mechanical properties, and toughness characteristics of ultra-high-performance concrete. The feasibility of using heavy-metal-sludge sintered slag as an internal curing material to partially replace fine aggregates in UHSC will be analyzed. Additionally, microstructural analyses using techniques such as XRD, TG, FTIR, and SEM will be conducted to explore the underlying mechanisms. Further evaluation of its feasibility as a replacement aggregate is needed, alongside providing technical support for the high-value utilization of heavy-metal sludge.

## 2. Materials and Methods

### 2.1. Raw Materials

Portland cement (P·II 52.5) was used in this study. The supplementary cementitious materials included silica fume and ground granulated blast furnace slag (GGBFS), while the fine aggregates consisted of river sand and sintered slag aggregate derived from heavy-metal sludge. A polycarboxylate-based high-range water reducer with a solid content of 35% was employed as the chemical admixture. The sintered slag aggregate was supplied by Yuyuan Ninghai Environmental Technology Co., Ltd. (Ningbo, China), while all other materials were provided by Guangtian Component Group Co., Ltd. (Ningbo, China). The fibers used were copper-coated straight steel fibers with a length of 13 mm, a diameter of 0.2 mm, and a tensile strength of 3015 MPa. The chemical compositions of the cementitious materials and sintered slag aggregate are presented in Table 1. As shown, the sintered slag aggregate contained a high combined content of SiO_2_ and Al_2_O_3_, reaching up to 78.3%, while its CaO content is significantly lower than that of the cement.

**Figure 1:**
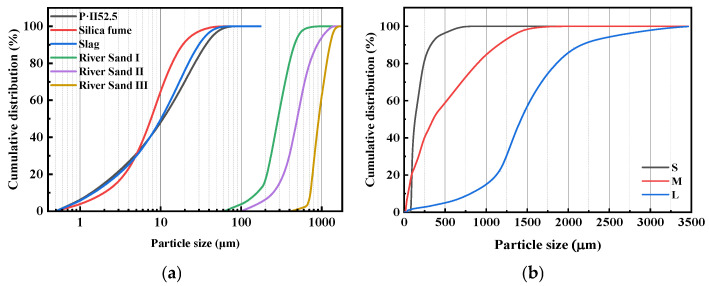
Particle size distributions of the experimental group: (**a**) cementitious materials and river sand; (**b**) sintered slag aggregates with different gradations.

**Figure 2:**
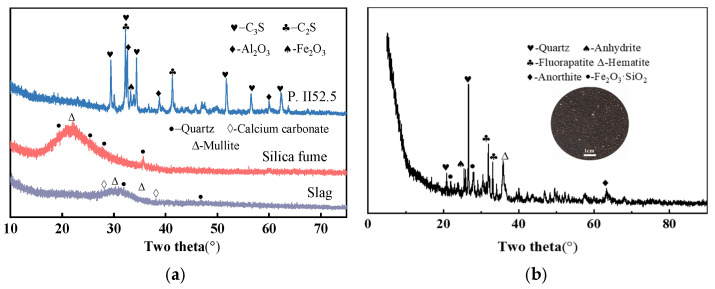
XRD patterns of raw materials: (**a**) cementitious materials; (**b**) sintered slag.

Figure 1a illustrates the particle size distributions of the cementitious materials and river sand. The primary particle size ranges were 5–30 μm for cement, 5–15 μm for silica fume, and 5–20 μm for GGBFS. Figure 1b presents the particle size distributions of the sintered slag aggregates with different gradations. The aggregates were sieved into three categories, fine (S), medium (M), and coarse (L), for use in subsequent experiments. Figure 2a shows the XRD patterns of cement, silica fume, and GGBFS. The main crystalline phases in the cement were identified as C_3_S, Al_2_O_3_, C_2_S, and Fe_2_O_3_. Figure 2b displays both the XRD patterns and morphological characteristics of the sintered slag aggregates, which primarily consisted of Fe_2_O_3_, SiO_2_, and Al_2_O_3_. Additionally, the apparent densities of cement, silica fume, and mineral powder were 3.042 × 10^3^ kg/m^3^, 2.298 × 10^3^ kg/m^3^, and 2.860 × 10^3^ kg/m^3^, respectively. The sintered slag particles exhibited an ellipsoidal shape, with a fineness modulus between 2.65 and 2.71, an apparent density of 1300 kg/m^3^, a bulk density of 900 kg/m^3^, and a water absorption rate of 8%.

**Figure 3:**
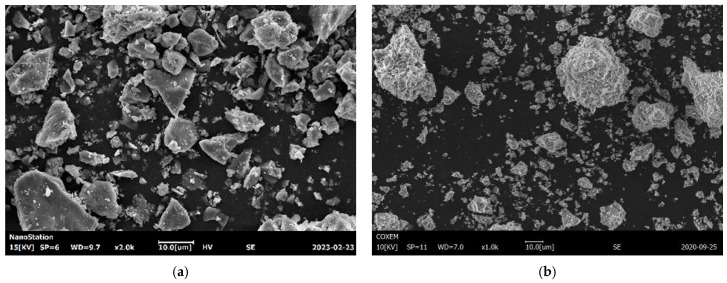
SEM images of P·II 52.5 and sintered slag: (**a**) cement; (**b**) sintered slag.

Table 2 presents the heavy-metal leaching concentrations of the sintered slag aggregates. The determination of heavy-metal leaching concentrations was conducted in accordance with the test method for leachable ions of heavy metals in cement mortar (GB/T 30810-2014 [18]). After the leachate was extracted, the concentrations of heavy metals were measured using inductively coupled plasma optical emission spectroscopy (ICP-OES). According to the limits specified in the “Technical specification for co-processing of solid waste in cement kiln” (GB/T 30760-2024 [19]), the slag aggregates fell within the category of general solid waste. To observe the composition and surface characteristics of the sintered slag aggregate, the microscopic morphologies of the cement and ground sintered slag samples are shown in Figure 3. The P·II 52.5 cement particles were smaller and uneven in size and had relatively smooth surfaces, while the sintered slag particles were larger and more irregular in size, exhibited rougher surfaces, and had higher specific surface areas.

### 2.2. Mix Proportions

Table 3 presents the mix proportions for the mechanical performance and microscopic mechanism tests of UHSC with different replacement rates and particle gradations of sintered slag aggregates. The volume fraction of steel fibers was 2%. The labels 10, 15, 20, and 25 in the numbering system represent sintered slag aggregate replacement ratios of 10%, 15%, 20%, and 25%, respectively. S, M, and L denote the use of sintered slag aggregates with fine, medium, and coarse particle gradations, respectively. The basic water-to-binder ratio was 0.18. With the increase in the replacement rate of sintered slag, additional water was added to compensate for the slag’s water absorption, thereby providing internal curing for the UHSC. UH0 represents the control group without the addition of sintered slag. In this study, additional water was added based on the original mix proportion to incorporate an internal curing agent, with the total mixing water being the sum of the original water content and the additional water. During the mixing process, the sintered slag aggregates were pre-wetted.

### 2.3. Testing Methods

#### 2.3.1. Test Method for Fluidity of UHSC 

UHSC was prepared according to the mix proportions in Table 3, and its fluidity was measured using the “Test method for fluidity of cement mortar” (GB/T 2419-2005 [20]) with the table flow test. The freshly mixed concrete was quickly filled into the truncated cone mold in two stages. The mold was then gently lifted vertically, and the table was immediately vibrated 25 times. After vibration, the diameters of the bottom surface of the concrete were measured in two perpendicular directions using calipers, and the average value obtained was considered the fluidity.

#### 2.3.2. Test Method for Mechanical Properties of UHSC 

UHSC was prepared according to the mix proportions in Table 3, and specimens with dimensions of 40 mm × 40 mm × 160 mm were cast. All formed specimens were covered with plastic film and placed in a room with a temperature of 20 ± 2 °C and relative humidity of 90 ± 5% for 24 h before demolding. After demolding, the specimens were stored in a standard curing room until the ages of 3, 14, 28, and 90 days. At each curing age, the compressive strength and flexural strength of the specimens were measured. All mechanical property tests were conducted using an electromechanical universal testing machine with a maximum load capacity of 200 kN. The loading rate for the flexural strength test was 60 N/s, while the compressive strength test was conducted at a loading rate of 2400 N/s. During the flexural test, a camera was set up to record the real-time displacement changes of the specimens under loading. The recorded data were analyzed using ORIGIN 2021 software to obtain the fracture curve for each specimen. The fracture energy was calculated by dividing the area enclosed between the fracture point and the X-axis by the cross-sectional area of the specimen [21]. The specific formula is shown in Equation (1).

(1) $$\;G_{f}=\frac{\int_{0}^{\delta_{0}}P\left(\delta \right)d\left(\delta \right)+mg\delta_{0}}{A_{lig}}=\frac{W_{0}+mg\delta_{0}}{hb}$$

where

*δ*_0_ is the deflection at the crack initiation point (mm);

*m* is the mass of the specimen (g);

*A_lig_* is the area of the fractured cross-section (mm^2^);

*h* and *b* are the width and height of the specimen cross-section (mm), respectively;

*W*_0_ is the crack initiation energy (N·mm).

#### 2.3.3. Test Method for Microstructural Analysis of UHSC 

Two molar ratio mixtures (UH0 and UH20L) were selected for microstructural analysis. For each mix ratio, a 50 mm × 50 mm × 50 mm sample was prepared for testing at 28 days. After curing, a portion of the samples was immersed in anhydrous ethanol at a concentration of 95 ± 1% for 48 h to stop hydration. After hydration cessation, the dried fragments were manually ground using a mortar and a pestle and sieved through a 0.02 mm sieve for XRD, FTIR, and TG analyses. In addition, after crushing the samples, the relatively smooth internal surfaces were gold-coated and subjected to SEM imaging for microstructural observation.

The XRD analysis was performed using a Bruker D2 diffractometer (Bruker AXS GmbH, Karlsruhe, Germany) with a scanning range of 10°–80° and a step size of 0.02°. TG analysis was carried out using a Pigaku Thermo Plus EVO2 thermal analyzer (Rigaku Corporation, Tokyo, Japan), with a nitrogen atmosphere and a temperature range of 30–1000 °C, at a heating rate of 20 °C/min. FTIR analysis was performed with a Nicolet iS10 spectrometer (Thermo Fisher Scientific, Waltham, MA, USA) in absorbance mode, with a wavenumber range of 500–4000 cm^−1^. In addition, SEM was conducted using a COXEM EM-30AX+ microscope (COXEM Co., Ltd., Daejeon, Republic of Korea)with a magnification of 2000×.

## 3. Results

### 3.1. Fluidity

**Figure 4:**
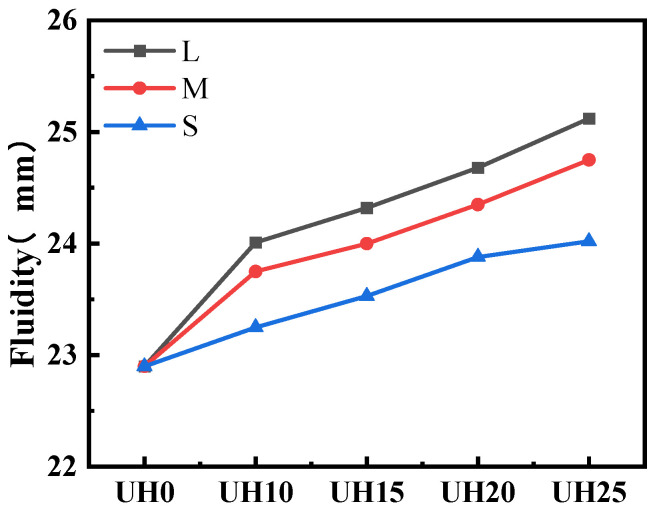
Effect of sintered slag replacement ratio and particle gradation on fluidity of UHSC.

Figure 4 shows the effect of sintered slag aggregate replacement ratio and particle gradation on the fluidity of UHSC. As can be seen from Figure 4, with the increase in the replacement ratio of sintered slag aggregates, the fluidity of UHSC gradually increases and remains higher than that of the control group (UH0). This is primarily due to the spherical shape of the sintered slag aggregates. The high sphericity of the lightweight aggregates reduces the inter-aggregate friction, facilitating the formation of a lubricating layer around the aggregates. This “ball-bearing” effect leads to a gradual increase in fluidity as the replacement ratio of sintered slag aggregates increases [22,23]. Furthermore, comparing the fluidity of UHSC with different particle gradations of sintered slag aggregates reveals that for the same replacement ratio, the fluidity increases as the particle gradation becomes coarser. This is because sintered slag aggregates with coarser gradation have a smaller specific surface area, thus requiring less paste to coat them. As a result, the paste in the concrete becomes more concentrated, and the excess paste increases the fluidity of UHSC [24]. Conversely, when the sintered slag aggregate has a finer gradation, the higher specific surface area requires more paste to fill the voids between the aggregates, leading to lower fluidity [25].

### 3.2. Mechanical Properties

(1) Flexural strength

**Figure 5:**
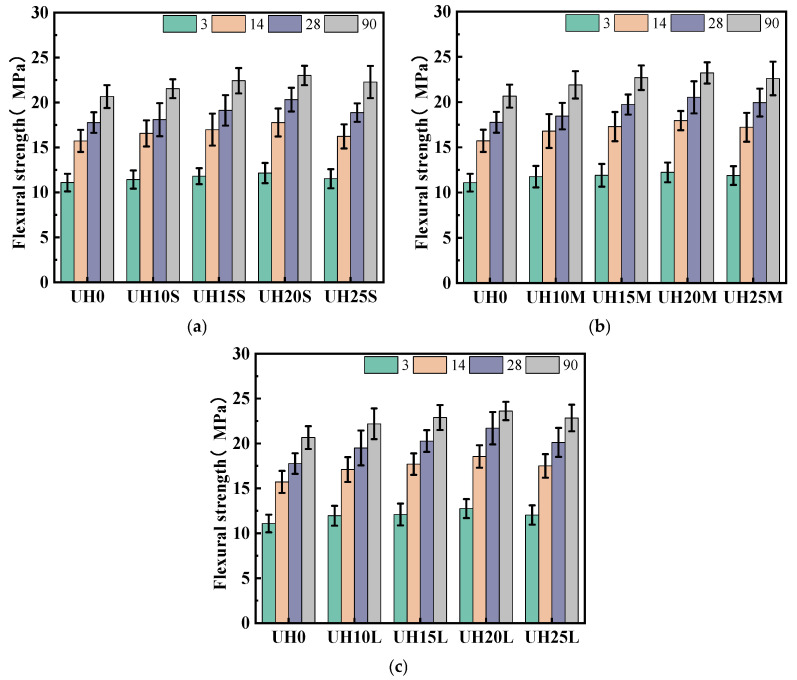
Effect of sintered slag aggregate replacement ratio on flexural strength of UHSC: (**a**) fine sintered slag aggregate; (**b**) medium sintered slag aggregate; (**c**) coarse sintered slag aggregate.

The effect of the replacement ratio of sintered slag aggregates on the flexural strength of UHSC is illustrated in Figure 5. With the increase in curing age, all specimens exhibit a steady rise in flexural strength. Figure 5a–c represent the flexural strength of the fine, medium, and coarse slag aggregate specimens, respectively. It can be seen that for the same particle gradation of sintered slag aggregates, the flexural strength of UHSC first increases and then decreases as the replacement ratio of sintered slag aggregates increases, with all values being higher than that of UH0. The experimental results indicate that the incorporation of sintered slag aggregates significantly improves the flexural strength of the UHSC specimens. The optimal flexural strength is achieved when the replacement ratio of sintered slag aggregates is in the range of 15% to 20%. The flexural strength of sintered slag aggregate UHSC increases rapidly and is consistently higher than that of UH0. This can be attributed to the low water-to-cement ratio of UHSC, which requires a significant amount of water for the hydration reaction. The moisture present in the sintered slag aggregates is released during the hydration process [26], providing sufficient water for hydration, thereby enhancing the flexural strength of concrete [15,27]. Additionally, the porous structure of the slag aggregate contributes to a micro-filler effect at appropriate dosages, improving the pore structure and overall compactness of the concrete. However, when the replacement level exceeds 25%, the excessive accumulation of slag particles may result in the formation of an interconnected pore network, ultimately leading to the deterioration of the concrete’s mechanical properties.

**Figure 6:**
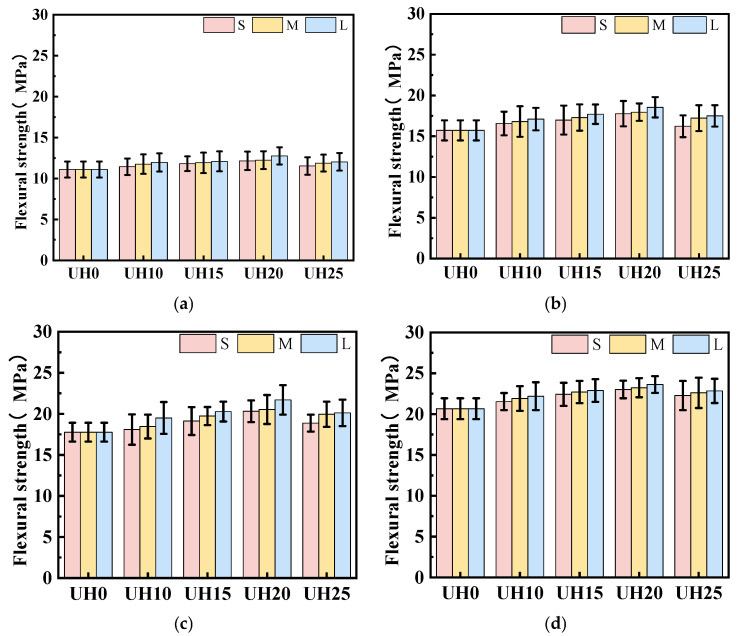
Effect of sintered slag aggregate particle gradation on flexural strength of UHSC: (**a**) 3d; (**b**) 14d; (**c**) 28d; (**d**) 90d.

Figure 6 illustrates the effect of sintered slag aggregate particle gradation on the flexural strength of UHSC. It can be observed that at a constant replacement ratio, the flexural strength increases progressively with coarser aggregate gradations. This trend highlights the critical role of particle gradation in determining the mechanical performance of UHSC. The improvement in flexural strength is primarily attributed to the porous and loosely structured nature of the sintered slag aggregates, which exhibit significantly higher water absorption than river sand. For sintered slag aggregates with different particle gradations, the thickness of the surface vitrification layer formed during sintering is roughly the same. Therefore, for coarser gradations of sintered slag aggregates, the proportion of the porous internal structure is larger, resulting in greater water absorption. As the hydration process progresses, the moisture content of UHSC decreases [28]. Under the influence of the moisture gradient force, coarser gradations of sintered slag aggregates release more moisture, which further promotes the continued reaction of the supplementary cementitious materials in the matrix, thereby enhancing the flexural strength of UHSC [29,30].

(2) Compressive strength

**Figure 7:**
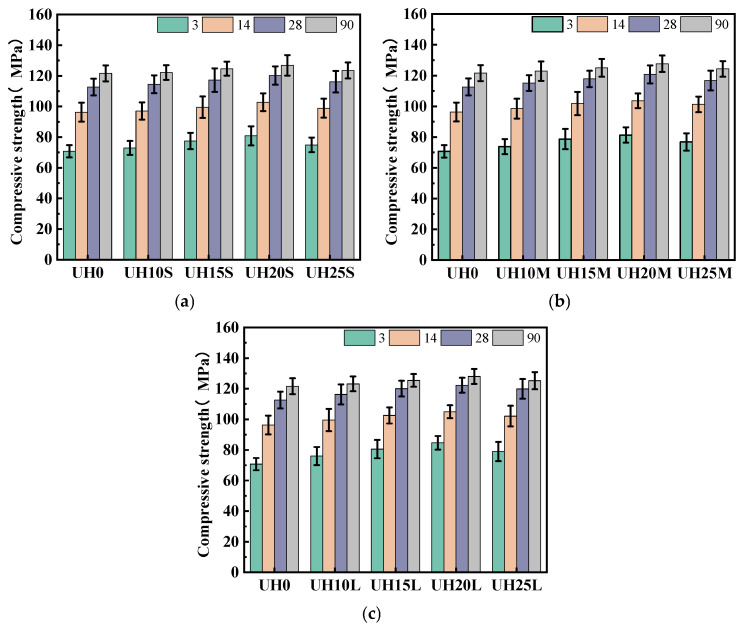
Effect of sintered slag replacement ratio on compressive strength of UHSC: (**a**) fine sintered slag aggregate; (**b**) medium sintered slag aggregate; (**c**) coarse sintered slag aggregate.

Figure 7 presents the effect of the sintered slag aggregate replacement ratio on the compressive strength of UHSC. As shown in Figure 7a–c, corresponding to fine, medium, and coarse gradations, respectively, the compressive strength of all specimens increases with the curing age. Notably, for each particle gradation, the compressive strength initially increases with the replacement ratio of sintered slag aggregates, reaching a peak at 15–20%, before declining at higher replacement levels. Nevertheless, all modified mixes outperform the control group (UH0) in terms of compressive strength. The experimental results indicate that the inclusion of sintered slag aggregates significantly contributes to the improvement in compressive strength in UHSC specimens. Among the various replacement ratios, when the replacement ratio of sintered slag aggregates is in the range of 15–20%, the compressive strength of the specimens is optimal. Higher replacement ratios of sintered slag aggregates lead to a decline in compressive strength. The increase in compressive strength of sintered slag UHSC is mainly due to two reasons: on one hand, the strength of UHSC is significantly influenced by the interfacial transition zone. The rough surface of sintered slag aggregates provides better bonding with the matrix, which improves the microstructure of the interfacial transition zone and results in a significant increase in compressive strength. On the other hand, sintered slag aggregates form a relatively strong and rigid skeleton inside the UHSC matrix. The interlocking force between aggregates is enhanced, and the anchoring effect of steel fibers between the aggregates and the matrix further strengthens the overall performance of the system, leading to an increase in compressive strength [31].

**Figure 8:**
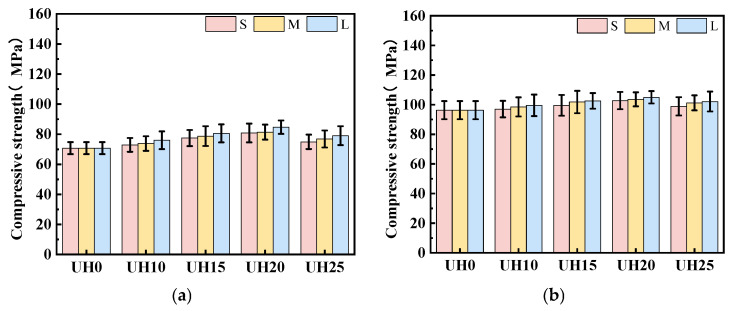

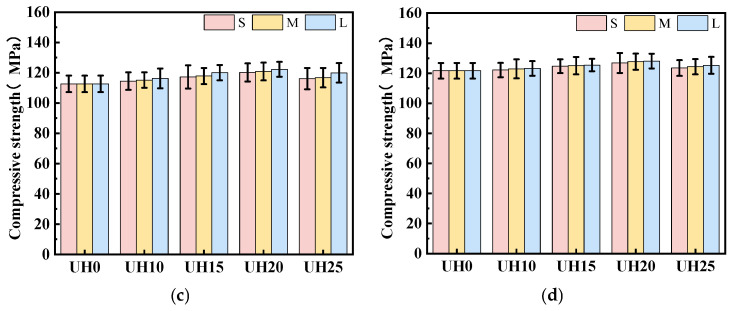
Effect of sintered slag particle gradation on compressive strength of UHSC: (**a**) 3d; (**b**) 14d; (**c**) 28d; (**d**) 90d.

Figure 8 illustrates the influence of sintered slag aggregate particle gradation on the compressive strength of UHSC. At a constant replacement ratio, an increase in particle gradation consistently leads to higher compressive strength. This trend highlights the significant role of aggregate size distribution in optimizing the mechanical performance of UHSC. The observed enhancement in strength with coarser gradations can be attributed to two main factors. First, finer aggregates present a larger specific surface area, which reduces the fluidity of the fresh mix and hampers effective compaction. This often results in weaker bonding at the aggregate–matrix interface and increased internal voids, ultimately compromising strength. Second, coarser aggregates promote stronger mechanical interlocking and provide larger contact areas with the matrix, which improves the interfacial bonding and contributes to a denser, more integrated microstructure. Together, these effects explain the superior compressive performance observed with the increase in particle size [31].

(3) Toughness characteristics

**Figure 9:**
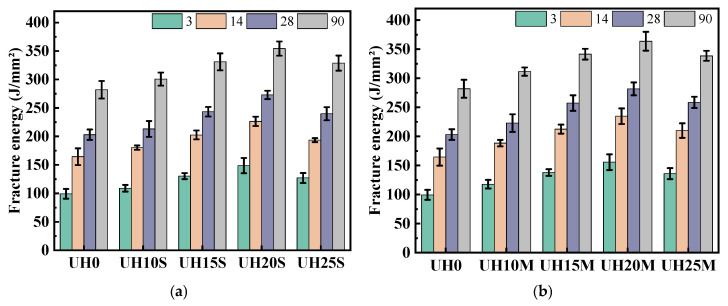

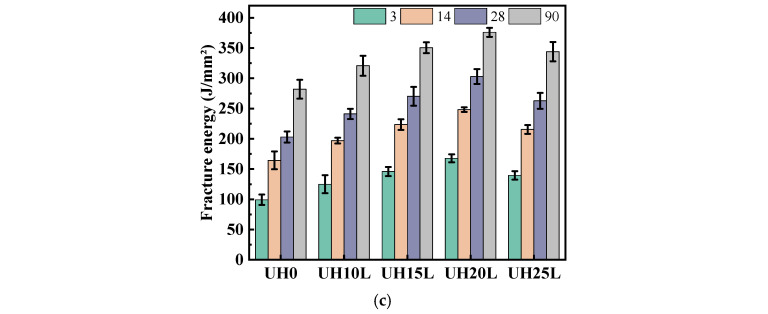
Effect of sintered slag replacement ratio on the fracture energy of UHSC: (**a**) fine sintered slag aggregate; (**b**) medium sintered slag aggregate; (**c**) coarse sintered slag aggregate.

Fracture energy reflects the energy absorbed by a specimen during fracture under applied force. The higher the fracture energy, the more the material can resist fracture or absorb energy during the loading process [32]. Figure 9 illustrates the impact of the replacement ratio of sintered slag aggregates on the fracture energy of UHSC. As shown in Figure 9, with the increase in curing age, the fracture energy of the specimens with different sintered slag replacement ratios and particle gradations increases gradually. Figure 9a–c present the fracture energy of fine, medium, and coarse sintered slag aggregates, respectively. From these figures, it can be seen that for the same particle gradation of sintered slag aggregates, the fracture energy of UHSC increases first and then decreases with the increase in the replacement ratio of sintered slag aggregates and is always higher than that of the baseline group, UH0. The experimental results indicate that the incorporation of sintered slag aggregates significantly improves the fracture energy of the UHSC specimens. Compared with other replacement ratios, the fracture toughness is optimal when the replacement ratio of sintered slag aggregates is in the range of 15–20%. A higher replacement ratio of sintered slag aggregates is detrimental to the fracture energy of the specimens. This can be attributed to the porous structure of sintered slag aggregates, which has unique stress–strain characteristics, including a large yield plateau in its stress–strain curve. When the concrete is subjected to external forces, the internal pore structure of the sintered slag aggregate gradually collapses and deforms. This process effectively absorbs and dissipates external impact energy [33,34].

**Figure 10:**
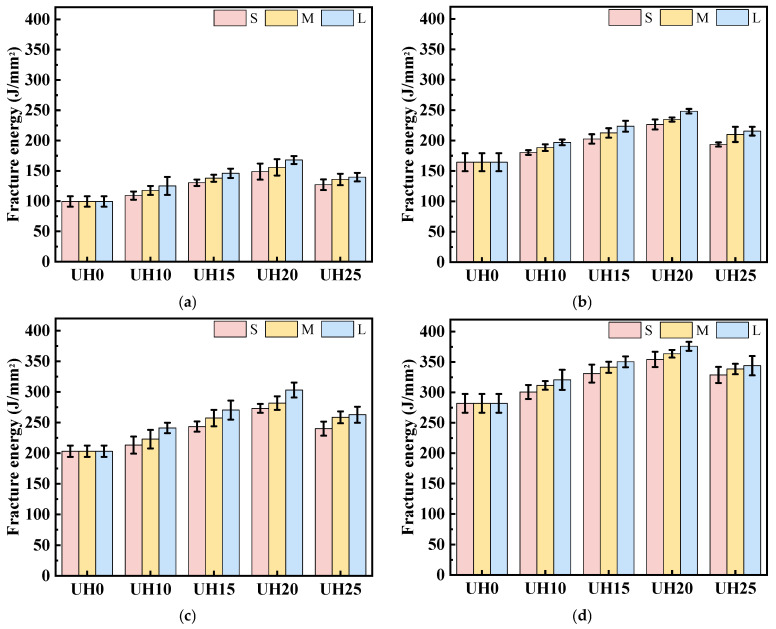
Effect of sintered slag particle gradation on fracture energy of UHSC: (**a**) 3d; (**b**) 14d; (**c**) 28d; (**d**) 90d.

Figure 10 shows the effect of the particle gradation of sintered slag aggregates on the fracture energy of UHSC. As seen in Figure 10, when the replacement ratio of sintered slag aggregates remains the same, the fracture energy of UHSC increases gradually with the increase in the particle gradation of sintered slag aggregates. The experimental results indicate that the particle gradation of sintered slag aggregates has a significant impact on the fracture energy of UHSC specimens. This is because increasing the particle gradation of sintered slag aggregates reduces the total surface area of the aggregates, allowing more cement paste to wrap around the surface of the steel fibers. As a result, the bonding ability between the matrix concrete and the steel fibers is enhanced, which improves the fracture energy of UHSC [35,36,37].

### 3.3. Microstructural Analysis

**Figure 11:**
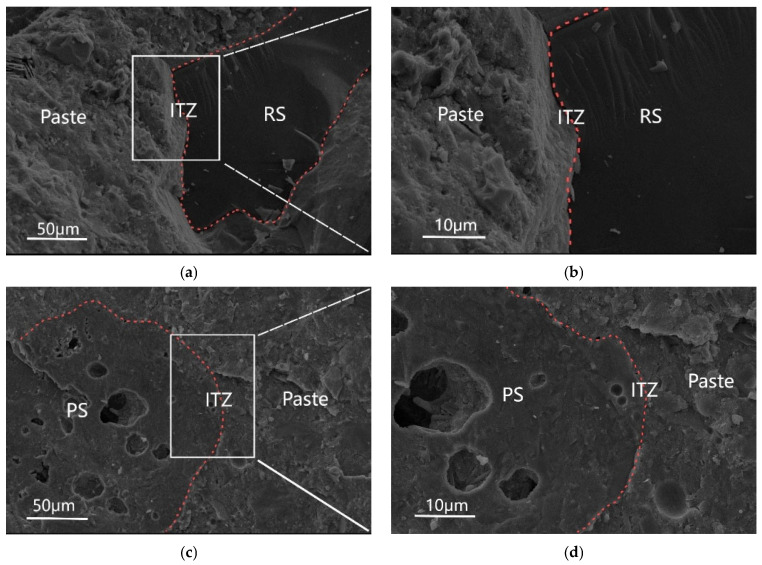
Effect of sintered slag on the microstructure of UHSC: (**a**) UH0 (1000×); (**b**) UH0 (2000×); (**c**) UH20L (1000×); (**d**) UH20L (2000×).

Figure 11 illustrates the influence of sintered slag aggregates on the microstructure of UHSC. Figure 11a,b show the microstructure of the interface transition zone (ITZ) between river sand (RS) and paste in the UH0 group at 28 days, magnified at 1000× and 2000×, respectively. Figure 11c,d show the microstructure of the ITZ between sintered slag (PS) and paste in the UH20L group at 28 days, magnified at 1000× and 2000×, respectively. Comparing the interface transition zones in both images, it can be observed that in the UH0 group, the interface between the river sand (a stronger aggregate) and the cement paste is compact with no obvious pores. The boundary between the aggregate and the cement paste is distinct. In contrast, the UH20L group shows that the sintered slag particles have a porous structure, consisting of irregularly shaped small pores and larger depressions. The bonding between the sintered slag and the cement paste is tight, with no obvious transition zone, and there are no visible cracks at the interface. This difference is attributed to the higher water absorption capacity of sintered slag, which eliminates the water film and layering effects caused by the “wall effect” at the interface during mixing [38]. Furthermore, a significant amount of hydration products can fill the pores on the rough surface of the sintered slag, causing the aggregate and paste interface to interlock without a distinct boundary. This strengthens the mechanical bonding at the interface, making the structure more compact [39,40].

**Figure 12:**
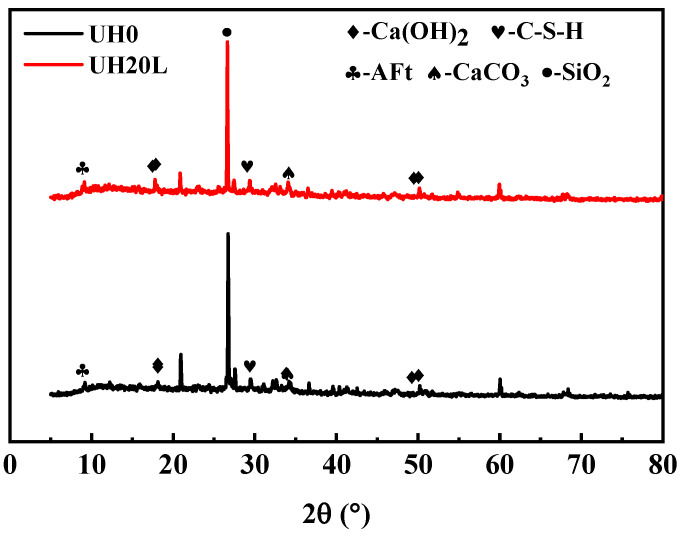
Effect of sintered slag on XRD patterns of UHSC.

Figure 12 shows the XRD patterns of UH0 and UH20L at 28 days. The crystalline phases were identified by comparing the diffraction patterns with standard reference data from the PDF-4+ database (International Centre for Diffraction Data, ICDD). From Figure 12, it can be seen that the main phases in the XRD patterns of both groups are SiO_2_, C-S-H, Ca(OH)_2_, and CaCO_3_ [41,42]. with a small amount of AFt. Among these, the diffraction peak of SiO_2_ is relatively high. From the diffraction peaks, it can be observed that UH20L has more Ca(OH)_2_ and a higher quantity of AFt compared with UH0. These phenomena indicate that the sintered slag aggregates promote the hydration of cement. The increase in Ca(OH)_2_ raises the pH level of the paste, creating an alkaline environment that disrupts the glass network structure of Al_2_O_3_ and SiO_2_. This facilitates the pozzolanic reaction between the reactive Al_2_O_3_ and SiO_2_ with Ca(OH)_2_, leading to the formation of C-S-H gels and other hydration products such as AFt. With the increase in hydration products, the density of UHSC improves, thereby enhancing its macro-mechanical properties [37].

**Figure 13:**
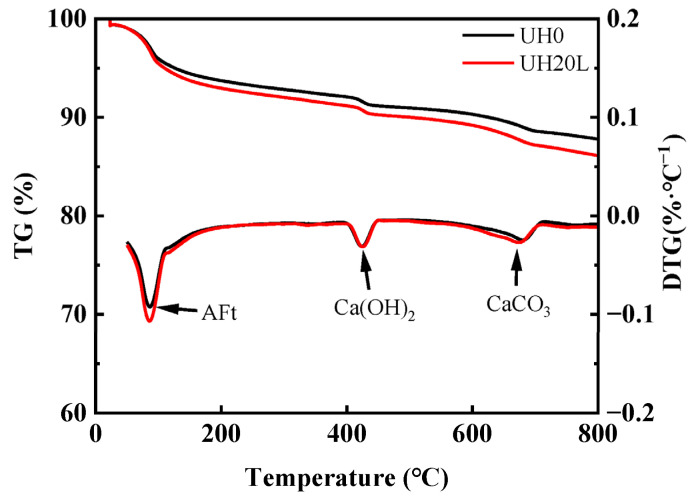
Effect of sintered slag on TGA of UHSC.

Figure 13 presents the TGA results of UHSC with sintered slag as a substitute for aggregate at 28 days. It can be observed that the TG curves of both groups exhibit similar trends with temperature changes, while the DTG curve reveals three prominent weight loss peaks corresponding to three sudden mass losses. The weight loss peak between 100 °C and 200 °C is attributed to the decomposition of C-S-H gel and AFt, and the peak between 400 °C and 500 °C is due to the dehydration of Ca(OH)_2_ crystals [43,44]. Within these two temperature ranges, the intensity of the weight loss peaks for UH20L is higher than that for UH0. This observation, combined with the mass loss data presented in the figure, indicates that UH20L produces more C-S-H gel, AFt, and Ca(OH)_2_ crystals. The comparison of the quantities of these three primary hydration products suggests that sintered slag significantly promotes the hydration reaction of UHSC, thereby increasing the amount of hydration products formed. The weight loss peak between 600 °C and 700 °C is caused by the decomposition of CaCO_3_ [45]. It is evident from the figure that the intensity of this peak for UH20L is slightly higher than that for UH0, indicating that UH20L contains more Ca(OH)_2_, which undergoes carbonation, leading to increased CaCO_3_ content. These findings are consistent with the phase diffraction peak changes observed in the XRD analysis.

**Figure 14:**
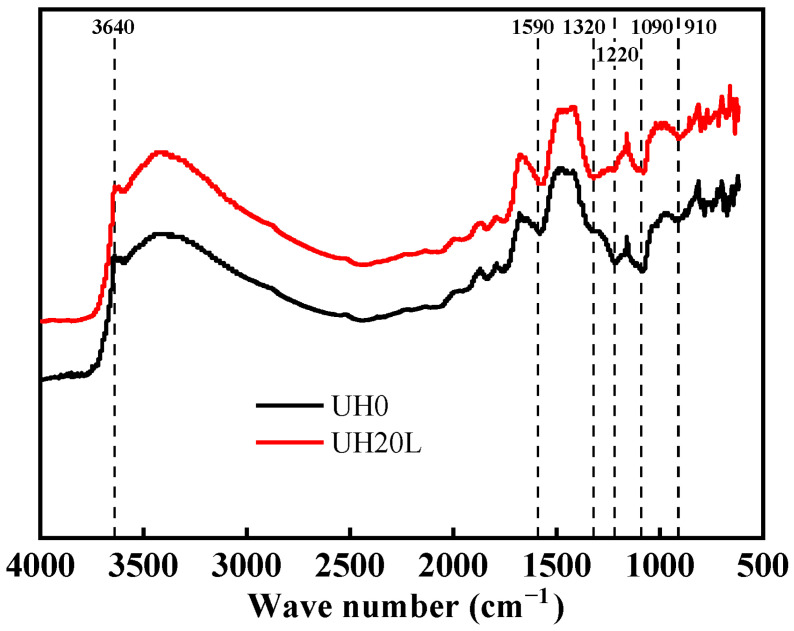
Effect of sintered slag on FTIR spectra of UHSC.

Figure 14 presents the FTIR spectra of UHSC specimens incorporating sintered slag as a partial replacement for aggregate at 28 days. As observed in Figure 14, the absorption peaks at approximately 910 cm^−1^, 1090 cm^−1^, 1220 cm^−1^, 1320 cm^−1^, 1590 cm^−1^, and 3640 cm^−1^ for the UH20L sample exhibit greater peak intensities and broader widths than those of UH0 [46]. The peaks at 910 cm^−1^ and 1090 cm^−1^ correspond to the stretching vibrations of Si-O bonds, which are characteristic of C-S-H gel. The decreased transmittance and the shift of these peaks toward higher wavenumbers in UH20L suggest an increased degree of polymerization of silicate anions and higher content of C-S-H gel as a result of sintered slag incorporation. The absorption features near 1090 cm^−1^ and 1220 cm^−1^, and 320 cm^−1^ correspond to the out-of-plane bending vibrations of CO_3_^2−^ and the symmetric stretching vibrations of C-O bonds, indicating the presence of CaCO_3_. These results imply that the addition of sintered slag enhances the formation of CaCO_3_. Moreover, the peaks at 1590 cm^−1^ and 3640 cm^−1^ are attributed to the bending and asymmetric stretching vibrations of H-O bonds, which are associated with the internal vibrations of crystal water—mainly originating from the hydration of C-S-H gel. The lower transmittance of these absorption bands in UH20L indicates a greater quantity of hydration products containing crystalline water. Overall, these findings confirm that the incorporation of sintered slag into UHSC promotes the formation of C-S-H gel and CaCO_3_ crystals, thereby reinforcing the observations derived from the XRD and TG-DTG analyses.

## 4. Conclusions

(1) As the replacement ratio of sintered slag for aggregates increases, the fluidity of UHSC shows a gradual increase. Coarse sintered slag exhibits the best effect on improving the fluidity of UHSC. This is because sintered slag has a spherical shape, and light aggregates with high sphericity reduce inter-aggregate friction. A lubricating layer is more easily formed around the aggregates, exhibiting a “ball-bearing” effect, which promotes the increase in the fluidity of UHSC. Additionally, when the sintered slag has a larger particle size, its specific surface area is smaller, requiring less cement paste for encapsulation, resulting in a more concentrated paste in concrete, and the excess paste improves the fluidity of concrete.
(2) Sintered slag can effectively enhance the flexural strength and compressive strength of UHSC. The enhancement follows a trend of an initial increase and then a decrease as the sintered slag replacement ratio increases. When the replacement ratio is in the range of 15–20%, the flexural strength and compressive strength of the specimens are optimal. Coarse sintered slag exhibits the best effect on improving both the flexural and compressive strength of UHSC. However, when the replacement ratio exceeds 20%, the excessive accumulation of slag particles results in the formation of an interconnected pore network, ultimately leading to the deterioration of the mechanical properties of concrete.
(3) From the perspective of fracture toughness, the toughness of UHSC is significantly improved with the incorporation of sintered slag. As the replacement ratio of sintered slag for aggregates increases, the fracture energy of UHSC initially increases and then decreases. The appropriate amount of sintered slag incorporation helps to improve the fracture energy of UHSC. When the replacement ratio is controlled within the range of 15–20%, the fracture energy of the specimens is greater. However, excessively high replacement ratios of sintered slag can adversely affect the fracture energy of the specimens. As the particle gradation of sintered slag increases, the fracture energy of the UHSC specimens shows a gradually increasing trend. This is because larger particles of sintered slag may improve the internal stress distribution of concrete, reducing local stress concentration and thus enhancing the material’s toughness.
(4) Microstructural morphology and phase composition analysis indicate that sintered slag primarily enhances the internal curing effect of UHSC by accelerating the cement hydration process. Sintered slag promotes the formation of a considerable amount of Ca(OH)_2_, C-S-H gel, and an appropriate amount of CaCO_3_ in concrete, which significantly improves the aggregate–cement interface transition zone. This helps to strengthen the mechanical bonding ability in the interface zone, leading to a more tightly bound particle interaction.

## Figures and Tables

**Table 1:** Chemical composition of cementitious materials and sintered slag.

Type	SiO_2_	Al_2_O_3_	Fe_2_O_3_	CaO	MgO	SO_3_	K_2_O	LOI
P·II 52.5	23.50	5.50	4.50	54.20	5.16	3.46	1.64	2.04
Silica fume	94.60	1.60	0.40	0.50	1.00	0.30	—	1.60
Slag	36.60	11.70	0.90	43.50	6.10	0.30	—	0.90
Sintered slag	57.45	20.85	4.92	6.09	4.64	2.96	3.09	0.00

**Table 2:** Leachate concentrations of heavy metals from sintered slag.

Material	Concentration (mg·L^−1^)			
	Ni	Cr	Zn	Cu
Sintered slag	0.162	0.066	0.325	0.065
Limited value	0.200	0.200	1.000	1.000

**Table 3:** Mix proportions for testing the effect of sintered slag on the mechanical properties of UHSC (kg/m^3^).

No.	P·II52.5	Silica Fume	Slag Powder	River Sand I	River Sand II	River Sand III	Sintered Slag	Water	Steel Fibers	Added Water
UH0	770	137.5	137.5	682.0	121.0	297.0	0	188	10	0
UH10S	770	137.5	137.5	587.6	104.3	297.0	95.2	188	10	7.6
UH15S	770	137.5	137.5	540.4	95.9	297.0	142.8	188	10	11.4
UH20S	770	137.5	137.5	493.2	87.5	297.0	190.4	188	10	15.2
UH25S	770	137.5	137.5	446.0	79.2	297.0	237.9	188	10	19.0
UH10M	770	137.5	137.5	613.8	109.0	267.4	95.2	188	10	7.6
UH15M	770	137.5	137.5	579.7	103.0	252.5	142.8	188	10	11.4
UH20M	770	137.5	137.5	545.6	96.9	237.7	190.4	188	10	15.2
UH25M	770	137.5	137.5	511.5	90.9	222.9	237.9	188	10	19.0
UH10L	770	137.5	137.5	682.0	121.0	190.0	95.2	188	10	7.6
UH15L	770	137.5	137.5	682.0	121.0	136.4	142.8	188	10	11.4
UH20L	770	137.5	137.5	682.0	121.0	82.9	190.4	188	10	15.2
UH25L	770	137.5	137.5	682.0	121.0	29.4	237.9	188	10	19.0

## Data Availability

The raw data supporting the conclusions of this article will be made available by the authors on request.
